# A new perspective on scoring children’s originality: a standards-based criterion-referenced assessment approach

**DOI:** 10.3389/fpsyg.2025.1545396

**Published:** 2025-06-13

**Authors:** Kadir Bahar, Carol June Maker

**Affiliations:** ^1^Department of Educational Psychology, University of Georgia, Athens, GA, United States; ^2^Department of Disability and Psychoeducational Studies, University of Arizona, Tucson, AZ, United States

**Keywords:** teaching for creativity, originality, creativity, standards-based assessment, measurement of originality, criterion-referenced assessment, measurement of creativity

## Abstract

In this paper, we proposed and discussed the use of a standards-based criterion-referenced assessment (CRA) approach in scoring originality with a specific focus on teaching for creativity. Throughout the paper, we supported our perspective with examples, particularly addressing evidence-based educational models that were designed to develop students’ ability to solve real problems in creative and effective ways in different domains. Given the importance of using proper assessment methods in fostering and monitoring learning, we believe such an approach might have significant implications for teaching practices and future research.

## Introduction

Nurturing originality in school children is essential to prepare them as problem solvers (Bahar et al., 2024). When students are taught to think in unique ways, they develop the ability to generate new ideas (Sternberg, 2003), approach problems creatively (Torrance, 1972a), and adapt when situations are uncertain or unfamiliar (Runco, 1999). Amidst an era of rapid technological changes and shifting societal needs, these skills are more important than ever. Further, being original helps students build confidence and find joy in expressing their unique perspectives (Cropley, 1992). It fosters curiosity and encourages them to take ownership of their learning (Piirto, 2011). By creating space for originality in the curriculum, educators prepare students not just for academic or career success, but also to bring fresh thinking and new solutions to the communities and environments they will one day influence (Maker et al., 2023a).

While most educators understand the critical role of nurturing creativity in the classroom, only a small number can incorporate it effectively into their regular teaching routines (Beghetto, 2010; Beghetto and Kaufman, 2010) because most educational systems are not designed to cultivate creativity; fostering students’ creativity rarely is regarded as a learning objective (Sternberg, 2015). Moreover, many structural barriers prevent teachers from integrating creativity into their teaching practices (Bullard and Bahar, 2023).

Assessment of originality often creates a significant barrier to incorporating it into the school curriculum. The challenge lies in its subjective and multi-layered nature as originality is not something that fits neatly into standardized testing formats. Educators are generally more comfortable with skills that can be measured objectively, which makes originality harder to make a priority. Policymakers and educators alike tend to shy away from including creativity and originality as a core goal because of the lack of universally accepted ways to assess it (Bahar and Maker, 2015). This leads to concerns about whether assessments would be fair, reliable, or practical.

Adding to the challenge is the heavy emphasis on standardized testing and measurable outcomes in many education systems. With so much focus on test scores, skills that are easier to quantify often take precedence, while more abstract ideas, like creativity and originality, are pushed to the sidelines. Teachers, too, may feel unprepared to assess originality, especially when clear guidelines or tools are missing (Bahar and Maker, 2020). All of this makes weaving originality into everyday teaching difficult. To address these problems, we need assessment strategies that strike a balance, which is something structured enough to be useful but flexible enough to reflect the true nature of original thinking. Without this balance, originality will not be an integral part of the curriculum.

Addressing these challenges, in this paper, we proposed and discussed the use of a standards-based criterion-referenced assessment (CRA) approach in scoring originality with a specific focus on teaching for creativity. Throughout the paper, we supported our perspective with examples, particularly addressing evidence-based educational models that were designed to develop students’ ability to solve real problems in creative and effective ways in different domains (Maker et al., 2015). Given the importance of using proper assessment methods in fostering and monitoring learning, we believe such an approach might have significant implications for teaching practices and future research.

## What is originality?

For over a century, researchers have been deeply interested in the concept of originality, particularly in the context of creativity (Cattell, 1903; Guilford, 1950; Thorndike, 1920; Torrance, 1972b; Wilson et al., 1953). At its core, originality often is defined as the ability to generate ideas that are truly novel (Wilson et al., 1953). It is closely tied to the uniqueness of ideas, methods, solutions, or other human products and ideas, especially when compared to existing ones (Goff and Torrance, 2002). Many scholars regard originality as the most essential element of creativity, recognizing its fundamental role in the process of creative thinking (Barron, 1968; Guilford, 1982; Lubart et al., 2013; Runco and Okuda, 1991; Wilson et al., 1953). It is also closely tied to innovation (Acar et al., 2017) because producing unique solutions to problems requires not only cognitive and psychological skills but also a blend of convergent and divergent thinking processes (Guilford, 1968, 1982; Lubart et al., 2013; Simonton, 2000).

Recent studies have highlighted that the ability to produce original ideas is not merely an innate ability but a skill that can be cultivated through targeted instructional strategies (Cotter et al., 2020; Bahar et al., 2021; Maker et al., 2022). For example, children participating in an art-based creativity training program, which incorporated emotional exploration and problem-finding techniques, were able to generate more original ideas (Cotter et al., 2020) than those who did not participate in the training program. Similarly, professional adults who engaged in art observation to identify emotional nuances showed sustained improvements in original thinking (Hoffmann et al., 2020). Both children and adults who participated in these programs showed measurable improvements in their ability to think creatively and generate original ideas, with some of these skills persisting over time.

In another series of studies, Bahar et al. (2021) investigated the impact of teachers’ implementation of the Real Engagement in Active Problem Solving (REAPS) teaching model on developing creative problem solving, with a specific focus on originality. Observing over 230 students and 18 teachers (Maker and Pease, 2021) in a public elementary school located in a multicultural metropolitan area in New South Wales, Australia, the researchers found that teachers can make a significant impact on students’ creative problem solving in mathematics (Bahar et al., 2021) and science (Maker et al., 2022) and development of the “rich, diversified associative network” (Lubart et al., 2013, p. 42) of knowledge (Maker et al., 2021) through a high level of fidelity of implementation of the REAPS model. Students of all levels of ability made significant gains in creative problem solving in math and science along with development of the knowledge structure that facilitates originality (Maker and Bahar, 2024). Altogether, these findings suggest that integrating instructional and developmental strategies into creativity instruction can enhance one’s ability to identify meaningful problems and generate original solutions, affirming that originality is not an innate talent but a skill that can be nurtured and developed (Bahar, 2022a, 2022b).

Given this context, as an important side note, in this paper, we operationally defined originality as the production of unique or uncommon responses relative to a given educational standard or criteria. Moreover, despite it is often referred as a core dimension of related constructs such as creativity, divergent thinking, and innovation, we purposefully distinguished “originality” from these broader terms. For example, from an assessment perspective, tools that measure creativity typically integrate scores for all or most of the divergent thinking dimensions (e.g., the Torrance Tests of Creative Thinking measure fluency, flexibility, originality, and elaboration comprehensively). In contrast, assessments specifically targeting originality often focus on the rarity of responses, independent of other aspects of divergent thinking skills such as fluency or elaboration. For example, an assessment of originality might score a student’s response higher if it is statistically rare compared to peer responses or predetermined learning criteria, regardless of the number or complexity of ideas produced.

## Most common scoring methods of originality

Children’s originality, as one of the most important aspects of creativity (Wilson et al., 1953), has been generally scored via three major scoring techniques: (a) norm-referenced assessment, (b) sample-based assessment, and (c) expert-referenced assessment. Creativity researchers continue to grapple with the challenge of identifying a universally accepted scoring technique, as each approach brings its own set of strengths and limitations (Mouchiroud and Lubart, 2001).

### Norm-referenced scoring

Norm-referenced scoring is a common method in creativity research for assessing originality by comparing an individual’s responses to those of a broader population. This approach operates on the premise that rare or unique responses reflect greater originality. By analyzing the frequency of specific ideas or solutions within a normative dataset, researchers can assign scores that reflect how distinct a participant’s contributions are relative to their peers. For instance, the Torrance Tests of Creative Thinking (TTCT; Torrance, 1974), the most commonly used divergent thinking test, has norm-referenced scoring to measure creative thinking (Lubart et al., 2013). While norm-referenced scoring provides a systematic framework for quantifying originality, it also has limitations. It often depends heavily on the context and sample size, as what is considered “original” may vary significantly across different groups or cultural settings. Additionally, it risks overlooking the subjective value or creative quality of responses that may not be statistically rare but are still innovative or impactful within a given context. Moreover, the normative 32data can be outdated over the years, so the test author should update the data frequently (Mouchiroud and Lubart, 2001). Despite these challenges, norm-referenced scoring remains a valuable tool for identifying patterns and trends in creative thinking.

### Sample-based assessment

Sample-based scoring of originality in creativity research involves evaluating the uniqueness of responses relative to a specific sample or group, rather than relying on predefined norms (Mouchiroud and Lubart, 2001). This method is based on the way creative ideas stand out within a particular context, such as a classroom, a cultural group, or a specific research cohort. By focusing on the rarity of responses within the sample itself, this approach offers a flexible and context-sensitive way to measure originality. It is particularly useful in studies in which cultural or situational factors may influence creativity, as it allows researchers to tailor their assessment to the group under study. However, sample-based scoring can be limited by the size and diversity of the sample, as smaller or less varied groups may produce less reliable indicators of originality (Plucker et al., 2011).

### Expert-referenced assessment

Expert-referenced scoring of originality in creativity research involves evaluating responses based on judgments made by individuals with specialized knowledge or experience in the relevant field (Baer, 2017). Unlike norm-or sample-referenced approaches, using this method, experts assess the novelty and appropriateness of ideas within a given context. This method leverages the nuanced understanding of experts to judge the novelty and uniqueness of responses. One of the most common forms of this technique is known as the Consensual Assessment Technique (CAT; Amabile, 1982, 1996), in which multiple experts independently rate creative products, and the consensus among their evaluations is used to determine originality (Baer and McKool, 2009). For example, in artistic or scientific domains, experts may rate the uniqueness of ideas based on their professional experience and understanding of the field’s standards. This approach is considered a reliable measure of creativity, as it reflects domain-specific standards and criteria (Amabile, 1996; Kaufman et al., 2013). However, it can be resource-intensive, requiring the recruitment and coordination of qualified experts, and may introduce subjectivity based on individual biases or varying interpretations of creativity. Despite these challenges, expert-referenced scoring remains one of the gold standard tools for assessing originality, particularly in complex or specialized domains where expert judgment is needed.

## A new perspective: standards-based criterion-referenced assessment approach

A standards-based criterion-referenced assessment (CRA) approach is evaluation of originality based on specific, pre-established criteria that were guided by teachers and curriculum standards, rather than comparing responses to a norm group or relying on expert consensus. When a scoring technique such as this is guided by academic standards in the curriculum, such as Common Core Standards, one can identify this method as ‘teacher guided standards-referenced scoring.’ In this method teachers may pre-establish the criteria or they can use concepts in the learning objectives or standards to evaluate the originality of student outcomes. Also, teachers do not need to reach or collect normative and sample-based data to evaluate originality in student outcomes. Additionally, teachers do not need to invite experts to the assessment process because they can assess student work by making comparisons with the preestablished criteria. Perhaps the most significant advantage of standards-based CRA is that it opens doors for teaching for creativity because the use of the method allows assessment of originality in everyday learning outcomes in any standards-based educational settings. Moreover, standards-based CRA bridges standards-based teaching and teaching for creativity, which have been considered two opposite directions in learning sciences.

Let us investigate a learning standard from an elementary mathematics curriculum and a word problem associated with this standard:

Standard: CCSS.MATH.CONTENT.1.OA.D.8: Determine the unknown whole number in an equation relating three whole numbers (For example, determine the unknown number that makes the equation true in each of the equations 8 +? = 11). This standard is taken from U.S. Common Core Standards (Grade 1 » Operations & Algebraic Thinking: Work with addition and subtraction equations).

Criterion: Use of addition or subtraction operations to solve problems.

A Sample Problem to Assess Learning: Use the numbers (2, 4, 6) to write correct equations.

Given that the learning standard listed above is focused on working with addition and subtraction equations, student responses that use these operations (e.g., 2 + 4 = 6, 4 + 2 = 6, 6–2 = 4, 6–4 = 2) can be considered correct but not original. A solution including a novel operation, representation, or algorithm that deviates from the learning standard (in this case it is subtraction and addition), can qualify for originality (e.g., 4÷2=6−4 , 2^4^ = 6 + 6 + 4).

Here is another learning standard from an elementary science curriculum and a problem associated with this standard:

Standard: ESS3.C: Human Impacts on Earth Systems: Things that people do to live comfortably can affect the world around them. But they can make choices that reduce their impacts on the land, water, air, and other living things. (This standard is taken from Next Generation Science Standards).

Criterion: Discussion of human impact on the land will include examples such as cutting trees to clear land for living and depleting resources to build big.

A Sample Problem to Assess Learning: List the scientific problems you can see in this picture.

Given that the examples listed in the criterion above are focused on cutting trees and depleting resources to build big buildings, student responses that are associated with these examples [e.g., having limited areas to live in big cities, no places for animals to live, and destroyed wildlife] can be considered correct but not original. A novel response that deviates from the examples discussed during the lesson can qualify for originality [e.g., greenhouse and global warming].

In fact, the use of CRA of creativity has a long history dating back to Torrance’s (1973) “Checklist of Creative Positives,” which is a rubric he devised to assist educators in selecting, guiding, and encouraging creatively gifted disadvantaged children. Since then, many criterion-referenced rubrics and questionaries have been developed to assess creative personality and behaviors (e.g., Creative Product Semantic Scale [CPSS]; Creative Behavior Inventory [CBI]; Creative Achievement Questionnaire [CAQ]; Creative Solution Diagnosis Scale [CSDS]). However, none of these were appropriate tools to assess creativity in domain specific academic learning in educational settings.

**Figure 1:**
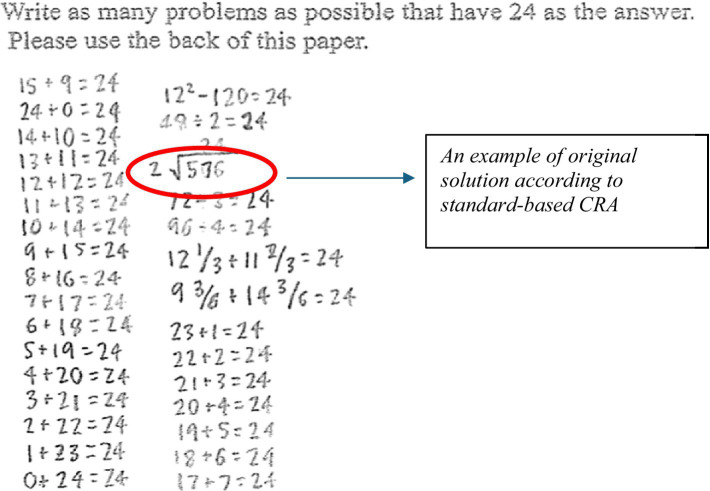
Example of an original solution to a mathematics problem. From “What does it take to be original? An exploration of mathematical problem solving” (Bahar et al., 2024). Reproduced with permission from Elsevier, with license number 6037631317699.

**Figure 2:**
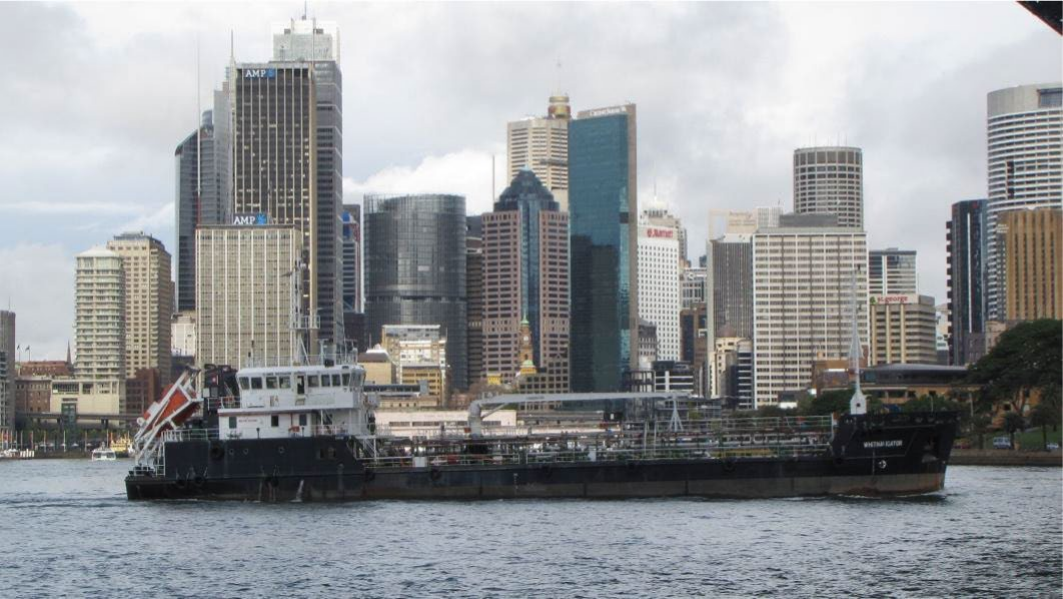
Picture used by teacher to assess originality in 4th grade science problem. From “Developing scientific, transformational, eloquent, artistic, mathematical, mechanical, emotional, relational, and social talents through problem solving: A conceptual, practical, evidence-based analysis” (Maker et al., 2023b). Reproduced with permission from Sage Publications, with order number 501990531.

Use of CRA in educational contexts with the goal of measuring growth in creative problem solving is limited; only a few studies included assessment tools that benefitted from criterion-referenced scoring (e.g., Maker, 2005). For example, in a study investigating originality in mathematical problem solving of elementary students, Bahar et al. (2024) assessed originality using a criterion-referenced scoring method and defined originality operationally as generating uncommon solutions to the mathematical problems in a way that included unique representations and/or creative use of operations and/or numbers. Scores on originality were assessed using open-ended problems in the math assessment portion of the DISCOVER assessment (see Figure 1). This scoring method is a measure of a test taker’s originality, depending on the grade level of a student, based on a standard set of criteria without reference to others. The scoring criteria included responses that had been documented by multiple experts considering the grade level content and standards in advance of the students’ assessment. For example, in the sample problem in Figure 2 students were asked to write as many problems as possible that have 24 as the answer. For this problem, producing at least one uncommon solution, such as use of fractions, decimals, roman numerals, symbolic representations, algebraic notations, and story was scored as original. A problem that included only basic facts and operations (e.g., 1 + 23 =?, 6 * 4 =?) was not original, whereas a problem that was different from these common problems was original.

This type of scoring method was found appropriate to assess children’s originality (Maker, 2005; Sak and Maker, 2006; Tan and Maker, 2020) because the list of uncommon/original solutions were derived by the experts considering the common/uncommon methods and solutions that were taught during that specific grade level of the test taker. These reports resulted in the development of categories/criteria of responses that could be considered original when scoring the mathematics assessment.

The use of criterion-based judgements of expert observers, such as those used in this assessment, share similarities with the CAT. In the first round of scoring of originality, two raters scored each solution individually for each student. Later, the raters discussed discrepancies between their scores for each student until they reached consensus. In contrast with the CAT, criteria to consider originality were available to the experts before reaching a consensus. Prior to reaching consensus, the inter-rater reliability coefficient for scores on originality was 0.81 (Bahar et al., 2024).

## Strengths of the approach

One advantage of standards-based CRA scoring is its objectivity and scalability (See Table 1). By adhering to a clear set of guidelines, variability that could arise from subjective judgment or differences in group norms is minimal. Use of guidelines makes this method particularly valuable in educational settings or large-scale studies in which consistency in scoring across different assessors is critical. Additionally, it provides clear feedback to participants, helping them understand how their creativity aligns with specific benchmarks. Moreover, this approach is particularly effective in structured assessments in which uniformity in evaluation is essential, as it ensures that every response is measured against the same criteria. We have presented a comparison summary in Table 1 that contrasts CRA with norm-referenced, sample-based, and expert-referenced methods in terms of scalability, fairness, subjectivity, and instructional alignment.

**Table 1:** Comparison of assessment methods.

Criteria	Criterion-referenced assessment (CRA)	Norm-referenced assessment	Expert-referenced assessment	Sample-based assessment
Scalability	High: Easily implemented across classrooms or districts using established criteria aligned with standards.	High: Widely applicable, depending on having current and representative normative data.	Low to Moderate: Limited due to reliance on expert judges and consensus processes.	Moderate: Scalable within defined groups or contexts; limited scalability across broader contexts due to context specificity.
Fairness	High: Evaluates students against clearly communicated, predefined criteria without peer comparison.	Moderate: Fairness varies; comparisons among peers might disadvantage specific student groups.	Moderate to High: Dependent on consistency among expert judgments; risk of bias through subjective judgments.	Moderate: Fairness contingent on sample composition; may disadvantage students unfamiliar with the cultural or contextual norms of the sample.
Subjectivity	Low to Moderate: Explicit criteria set within a large educational setting reduce subjectivity, though teacher-created criteria might introduce institutional bias.	Low to Moderate: Objective statistical scoring minimizes subjectivity; however, interpretations vary by context or sample size.	High: Depends extensively on subjective expert judgments, though consensus methods like CAT can mitigate this somewhat.	Moderate to High: Subjectivity arises from interpretations of rarity or uniqueness within smaller, specific contexts or samples.
Instructional Alignment	High: Direct alignment with curriculum and instructional objectives, facilitating targeted teaching strategies.	Moderate to Low: Less aligned with specific curriculum standards, focusing instead on statistical comparison of peer responses.	Moderate: Potentially strong domain-specific alignment; general alignment with broader curricular standards less explicit.	Moderate: Alignment dependent on the chosen context or sample; effective within specific classrooms or groups but less stable across broader curricular frameworks.

Additionally, incorporating standards-based CRA into teaching has significant implications for curriculum design and instructional practices. With this approach, educators can create learning activities and assignments designed to explicitly encourage students to engage in creative thinking and problem-solving, aligned with the originality criteria being assessed. This instructional alignment ensures that students are given intentional opportunities to practice and develop their creative abilities. Furthermore, the consistent use of such assessments can help teachers recognize patterns in students’ creative development, enabling more targeted interventions and support (Maker and Pease, 2021). Over time, this focus on originality can cultivate a culture of innovation within schools, preparing students to think critically and creatively in addressing real-world challenges. Ultimately, standards-based CRA in the assessment of originality provides a structured yet flexible framework for fostering and evaluating one of the most essential skills in the 21st century: creativity (Maker et al., 2021).

## Potential limitations of the approach

Despite its strengths, this approach is not without limitations. The rigid nature of predefined criteria can sometimes cause evaluators to overlook the contextual aspects of originality, particularly in highly subjective or culturally specific domains. A creative idea that might be groundbreaking in one context could be undervalued if it does not align with the predetermined standards. Moreover, defining the criteria themselves can be challenging, as originality is inherently context dependent. Specifically, teacher-created criteria might introduce institutional bias and subjectivity, whereas explicit criteria set within a large educational setting reduce subjectivity. Perhaps, one approach to mitigate this problem could be providing training to teachers who plan to design their own criteria or rubrics, specifically addressing strategies for minimizing bias. Another effective strategy might be for schools and districts to establish collaborative teams composed of expert teachers and curriculum designers, rather than relying solely on individual teachers. Such consortium-based criteria development could significantly reduce potential biases.

Another significant point is that norm-referenced scoring provides insights into how an individual’s originality stands out within a peer group, which is often a critical aspect of creativity research. Meanwhile, CRA does not offer this comparative perspective, making them less informative in studies aimed at understanding relative original performance. Similarly, by focusing on meeting specific criteria, this approach can oversimplify the evaluation of originality, reducing it to a checklist rather than capturing the broader, qualitative aspects of creative thinking. Predefined benchmarks may fail to capture the full range of creative expression, particularly in unconventional or unexpected responses. Norm-referenced scoring, by incorporating comparisons to a wider range of responses, often provides a richer understanding of originality.

Another important limitation is related to the potential overlap between mathematical knowledge and the appearance of originality. Indeed, when assessing originality in subject-specific contexts such as mathematics, it becomes essential to disentangle originality from content mastery. A student with advanced mathematical knowledge may naturally generate responses that appear more original because they have access to a broader range of strategies or operations (e.g., division, multiplication, or even algebraic notation). This does raise a valid concern about potential bias: are we assessing originality, or simply rewarding advanced content knowledge?

In fact, we believe that should be addressed not as a limitation of standards-based CRA, but rather as a call for intentional design and thoughtful rubric development to ensure fairness and validity. Having said that, we propose two considerations to address this call. First, criteria for originality in a standard-based CRA framework should be grade-level sensitive and grounded in curricular expectations. What counts as “original” for a third grader may not be the same for a fifth grader, and assessments should take developmental appropriateness into account (Sak and Maker, 2006). Second, educators can design tasks that level the playing field, for example, using non-routine, open-ended problems that do not favor specific procedural knowledge but instead prompt students to explore multiple pathways or representations. By designing tasks where multiple solution strategies are equally accessible, we can better isolate and reward original thinking rather than knowledge mastery alone (Leikin and Lev, 2007).

Finally, we want to point to a potential limitation related to international generalizability, as some readers might question if this approach is applicable for those working outside the U. S. or non-standardized educational contexts. Although the context we described, including the examples and applications of standards-based CRA, are drawn predominantly from U. S. standards (e.g., Common Core), we think this approach might be adapted to any educational setting that evaluates student learning based on any type of criteria, not necessarily a universal learning standard. Furthermore, most of the empirical support coming for this approach (e.g., DISCOVER and REAPS models), have been implemented in different countries including, Australia, Taiwan, and United Arab Emirates, providing support for the successful implementation of the approach across cultural and multilingual settings. Therefore, we do not anticipate educators outside the U. S. or those working in non-standardized educational contexts might have difficulties to adapt or implement the CRA effectively.

Despite these challenges, standards-based CRA offers a robust and practical tool in creativity research, especially when combined with other methods such as norm-referenced, sample-based, and expert-referenced scoring (See Table 1 for detailed comparison of assessment methods). Together, these approaches can offer a more comprehensive understanding of originality by balancing structured evaluation with flexibility to capture the depth and complexity of creative thinking (Maker et al., 2021; Maker et al., 2023a).

## Directions for future research

Future researchers can compare the predictive validity of scoring methods across various techniques, such as norm-referenced scoring and CAT. By examining how these techniques predict outcomes like creative problem-solving skills, academic performance, or real-world innovation, researchers can determine the relative strengths and weaknesses of each approach. This comparative analysis could provide valuable insights into which scoring methods are most effective for assessing originality in different contexts, thereby guiding educators and researchers in selecting the most appropriate evaluation tools.

Another possible direction for future research might focus on the development and validation of criteria across disciplines. Such exploration might be needed to understand broader application of this approach, which might provide educators with proper use of the technique across different disciplines. Beyond this, exploring teachers’ perceptions of using standards-based CRA for originality could reveal crucial information about its practical implementation and impact in educational settings. Teachers play a pivotal role in fostering creativity in students, and their acceptance of and confidence in such techniques can significantly influence their application (Bahar, 2022a; Beghetto, 2010). Researchers could investigate whether teachers find this scoring method easier to implement, fairer, or more aligned with fostering creativity when compared to traditional approaches. Understanding their concerns also would be valuable, such as the potential for subjectivity or added workload. Addressing these perceptions through future research could lead to the development of professional development programs or refinements in scoring frameworks, ensuring that standards-based CRA is both effective and widely embraced in fostering originality among students.

Relatedly, we recommend researchers to explore student of the fairness, clarity, and motivational impact of CRA. While much of the existing literature focuses on the instructional alignment of CRA, student voice remains an underexamined dimension. Understanding how students interpret and respond to CRA—particularly in terms of perceived fairness and transparency—could yield critical insights into its practical effectiveness and influence on student engagement and learning outcomes. Incorporating this perspective would not only provide a more holistic evaluation of CRA but also inform the development of assessment practices that are both pedagogically sound and student-centered.

## Final thoughts

For years, educators and researchers have expressed concern about the diminished focus on originality within educational systems. Despite its importance in fostering critical thinking and problem-solving skills, originality often remains underemphasized in curricula in which standardized testing and rote learning are the priorities (Bahar, 2022a; Beghetto and Kaufman, 2010). This oversight has sparked calls for a more balanced approach in which creative development is integrated with traditional teaching of academic subjects, aiming to cultivate well-rounded individuals equipped to navigate the complexities of the modern world (Maker and Bahar, 2024).

While it’s encouraging that creativity is now widely seen as a skill that can be taught and assessed, it’s important to remember that implementation of an effective assessment method would not be sufficient to foster originality in the classroom. Vygotsky (1978) reminds us that learning does not happen in isolation; it’s shaped by social interactions, tools, language, and cultural context. From this angle of view, originality is not just about generating novel ideas independently—it also involves building on others’ ideas, using cultural tools in new ways, and engaging in collaborative meaning-making (Sawyer, 2012). In other words, similar to creativity, originality is co-constructed. This means that when we assess students’ original work, particularly through CRA approaches, we should consider how students leverage their social and cultural resources. Doing so allows us to create assessments that are not only more inclusive and developmentally appropriate, but that also reflect the real, relational nature of how originality emerges in classrooms (Glăveanu, 2014). These perspectives challenge more individualistic or purely cognitive models of creativity and originality and encourage us to think more expansively about what it means to foster and assess creative potential in diverse learning settings.

In conclusion, adopting a standards-based CRA to score children’s originality may be a valuable shift in educational practice. Unlike traditional methods, including norm-referenced, sample-based, and expert-referenced techniques, this technique allows for individualized evaluation that emphasizes students’ individual creative potential rather than comparison with peers. By setting standard-based benchmarks for originality, educators can effectively identify and nurture creative abilities and also have the opportunity to develop their unique ideas. Additionally, this approach is aligned with models that promotes innovation and critical thinking in 21st-century education, with a goal to equip students with skills that are essential for success in an increasingly complex world (Bahar, 2023; Bahar and Maker, 2011; Maker et al., 2023a). Future research and teacher education will be critical in refining the implementation of this method, addressing potential challenges, and ensuring its wide acceptance. Ultimately, standards-based CRA is a promising framework for fostering a more inclusive and meaningful assessment of creativity in educational settings.
